# Abnormal decrement on high-frequency repetitive nerve stimulation in congenital myasthenic syndrome with GFPT1 mutations and review of literature

**DOI:** 10.3389/fneur.2022.926786

**Published:** 2022-09-15

**Authors:** Ran An, Huijiao Chen, Song Lei, Yi Li, Yanming Xu, Chengqi He

**Affiliations:** ^1^Department of Rehabilitation Medicine, West China Hospital, Sichuan University, Chengdu, China; ^2^Key Laboratory of Rehabilitation Medicine in Sichuan Province, Chengdu, China; ^3^Department of Pathology, West China Hospital, Sichuan University, Chengdu, China; ^4^Department of Neurology, West China Hospital, Sichuan University, Chengdu, China

**Keywords:** GFPT1, congenital myasthenic syndrome, tubular aggregates, RNS, repetitive nerve stimulation, high-frequency

## Abstract

**Objectives:**

Congenital myasthenic syndrome (CMS) is a clinically and genetically heterogeneous group of inherited disorders characterized by neuromuscular junction defects. Mutations in GFPT1 have been shown to underlie CMS. An increasing number of patients with CMS due to mutations in GFPT1 have been reported. However, a comprehensive review of clinical and genetic analyses of GFPT-related CMS worldwide is lacking, especially, given that the common or hotspot mutations in GFPT1 have not been reported. Here, we described the clinical and genetic findings of three patients with GFPT1 mutations from southwestern China and reviewed the clinical and genetic features of patients with GFPT1-related CMS worldwide.

**Methods:**

Clinical, laboratory, electrophysiological, myopathological, and genetic analyses of three patients with GFPT1-related CMS from southwestern China were conducted, and a review of previously published or reported cases about congenital myasthenic syndrome with GFPT1 mutations in the PubMed database was made.

**Results:**

The clinical, laboratory, electrophysiological, and myopathological features by muscle biopsy of three patients with GFPT1-related CMS were consistent with those of previously reported patients with GFPT1 mutations. Additionally, an abnormal decrement in high-frequency RNS was found. Two different homozygous missense mutations (c.331C>T, p.R111C; c.44C>T, p.T15M) were detected by whole-exome sequencing (WES) or targeted neuromuscular disorder gene panels.

**Conclusion:**

A distinct decremental response to high-frequency RNS was found in three patients with GFPT1-related CMS from southwestern China, which has never been reported thus far. In addition, the location and degree of tubular aggregates (TAs) seemed to be associated with the severity of clinical symptoms and serum creatine kinase levels, further expanding the phenotypic spectrum of GFPT1-related CMS. Lastly, some potential hotspot mutations in GFPT1 have been found in GFPT1-CMS worldwide.

## Introduction

Congenital myasthenic syndromes (CMSs) comprise a group of genetically and clinically heterogeneous inherited disorders affecting the transmission of signals at the neuromuscular junctions. The hallmark of CMS is fatigable and fluctuating weakness. The disease onset usually occurs from birth to the first 2 years of age, although patients can present much later in life. Mutations in a series of genes encoding post-, pre-, or synaptic proteins at the neuromuscular junction (NMJ) have been associated with CMS. Over 30 genes have been identified to date (1, 2). Limb-girdle congenital myasthenic syndrome (LG-CMS), a subtype of CMS, mostly inherited in autosomal recessive traits, is characterized by prominent fatigable weakness of the shoulder and pelvic girdle muscles, with subtle or no ocular and facial involvement (3).

GFPT1, encoding glutamine-fructose-6-phosphate transaminase 1, is the first and rate-limiting enzyme in the hexosamine biosynthetic pathway, the end product of which is uridine diphosphate-N-acetylglucosamine (UDP-GlcNAc), which serves as a common precursor for all amino sugars used for the synthesis of glycoproteins, glycolipids, and proteoglycans (4). Various proteins in the NMJ, such as muscle-specific kinase (MuSK) and acetylcholine receptor (AChR) subunits, are heavily glycosylated (4, 5). GFPT1 mutations contribute to an impairment of neuromuscular transmission by a defect in glycosylation in these proteins (4, 6, 7). Mutations in GFPT1 have been shown to underlie LG-CMS. LG-CMS patients with GFPT1 mutations usually share several common clinical features, including prominent fatigable weakness of proximal limb muscles, with no or minimal involvement of ocular, facial, bulbar, or respiratory muscles, normal or slightly elevated serum creatine kinase levels, electrophysiological evidence of NMJ dysfunction, mostly with the presence of tubular aggregates (TAs) in myofibers on muscle biopsies, and a good response to treatment with acetylcholinesterase inhibitors or 3,4-diaminopyridine (1–3, 8). To date, an increasing number of patients with LG-CMS due to mutations in GFPT1 (locus 2p13.3) (OMIM: 610542) have been reported from different countries and ethnic populations (6–27). However, clinical and molecular genetic analyses of GFPT-related LG-CMS worldwide are lacking, and common or hotspot mutations in GFPT1 have not been reported.

Here, in our study, we described the clinical, laboratory, electrophysiological, and myopathological findings in muscle biopsy and molecular genetic analysis of three unrelated patients with GFPT1-related CMS from southwestern China. Then, we reviewed the clinical and molecular genetic features of patients with GFPT1-related CMS from different countries and ethnic populations to identify common or hotspot mutational variants in GFPT1.

## Patients and methods

### Patients and clinical examinations

Three patients (two male, one female; aged 14, 15, and 32) from three unrelated Chinese families, suffering from a predominant limb-girdle pattern of muscle weakness and fatigability, were admitted to our hospital. Detailed medical history and results of neurological examinations were collected by two physicians specializing in neuromuscular disorders. Electrophysiological investigations, including standard electromyography (EMG), sensory and motor nerve conduction velocities, and repetitive nerve stimulation (RNS) at low-frequency (3, 5 Hz) and high-frequency (10, 20, and 50 Hz), were performed using standard techniques in each patient. During the high-frequency RNS, first, different painful tolerance or threshold value of three patients was considered. Additionally, from a clinical perspective, the amplitude of attenuation was positively related to the frequency of stimulation to some extent. Therefore, an increasing stimulation frequency was given in clinical practice. Considering the above factors, each patient may be given a different stimulation frequency at last (10, 20, or 50 Hz). Testing of acetylcholine receptors (AChRs) and muscle-specific tyrosine kinase (MuSK) antibodies was available in two patients. Ophthalmological or hearing examinations were performed on two patients. Also, other routine laboratory tests, including complete blood count, liver and renal function, serum glucose and lipid, serum electrolytes, serum creatine kinase, thyroid function, autoimmune function, electrocardiogram (ECG), chest computed tomography (CT), and echocardiography were conducted.

### Analysis of muscle biopsy

A left biceps brachii muscle biopsy was performed on three patients. Fresh muscle specimens were snap frozen in isopentane cooled in liquid nitrogen and 8-micron serial cross-sections were cut in a cryostat. Sections were stained with hematoxylin and eosin (H&E), modified Gomori trichrome stain, NADH-tetrazolium reductase (NADH-TR), succinic dehydrogenase (SDH), cytochrome oxidase (COX), adenosine monophosphate deaminase (AMP), myofibrillar ATPase at pH 10.4, 4.7, 4.4, PAS, Sudan black, and acid phosphatase. A portion of the muscle was also submitted for electron microscopic examination (EM), which was fixed in phosphate-buffered 2.5% glutaraldehyde, dehydrated, and embedded in resin.

### Molecular genetic analysis

After written informed consent from patients or their legal guardians was obtained, peripheral blood samples of our two patients (and their biological parents when available) were collected, and their genomic DNA was isolated from blood samples by the standard phenol-chloroform procedure. Then, mutational variants were screened by whole-exome sequencing (WES) in two patients (patients 1 and 2). Mutational genetic analysis of patient 3 was conducted by targeted neuromuscular disorder gene panels in another well-known tertiary medical center (detailed sequencing data were unavailable). Sanger sequencing was used to confirm the mutations detected in the proband, their parents, or relatives (primers, PCR, and Sanger sequencing conditions are available on request).

### Standard protocol approvals and patient consent

The present study was approved by the Institutional Ethics Committee of West China Hospital of Sichuan University (approval 2020-842). Written informed consent was obtained from the patients or patients' parents for participating in the study and publication of this paper. Additionally, a review of previously published or reported cases of congenital myasthenic syndrome with GFPT1 mutations in the PubMed database was made.

## Results

### Clinical data

All three patients were presented with predominant weakness of proximal limb muscles with fatigability and fluctuation, without the involvement of ocular, facial, bulbar, or respiratory muscles. No lordosis, joint contracture, muscle atrophy, or cognitive dysfunction was observed in any of them. Patient 1 was born from consanguineous parents, with two younger sisters, one younger sister having similar symptoms of fatigable and fluctuating muscle weakness and cataract, and the other having cataract only. Ophthalmological examinations were conducted in patient 1, revealing no abnormalities of the cornea, crystalline lens, or fundus. The ocular fundus, pure tone test, and auditory impedance examinations of patient 2 were also normal. The clinical symptoms and signs were the most serious in patient 1, moderate in patient 2, and mildest in patient 3.

### Laboratory findings

The serum creatine kinase level was mildly elevated in patient 1(335 IU/L, normal range:19–226), normal in patient 2 (203 IU/L, normal range: 19–226), and patient 3 (87 IU/L, normal range: 20–140). Testing of anti-AChRs and anti-MuSK antibodies in patient 1 and patient 2 were both negative.

### Electrophysiological findings

**Figure 1:**
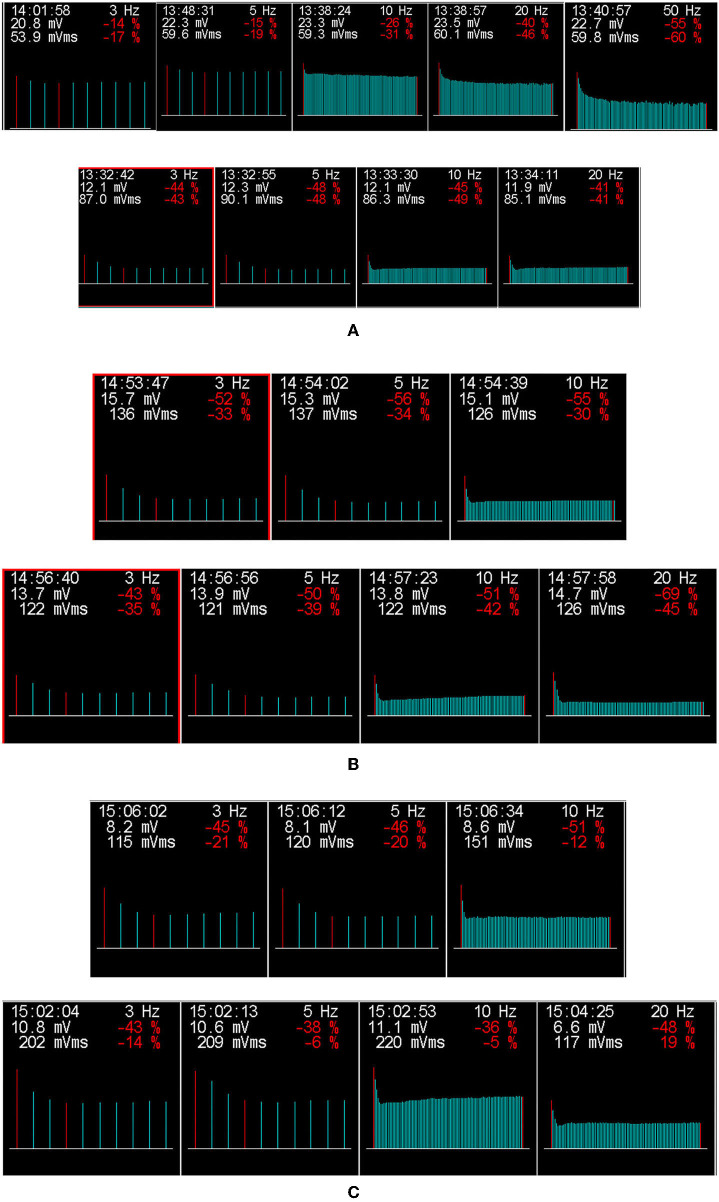
The results of low-frequency and high-frequency repetitive nerve stimulation (RNS) for three patients with GFPT1-related CMS from southwestern China. The amplitude of compound muscle action potential (CMAP) on RNS at both low-frequency (3, 5 HZ) and high-frequency (10, 20, and 50 Hz), which decreased by more than 15 and 30%, respectively, are considered abnormal. A dramatic decrement in the right abductor hallucis (top row) on RNS at 5, 20, and 50 Hz and in the right trapezius (bottom row) at 3, 5, 10, and 20 Hz stimulation was observed in patient 1 **(A)**. Additionally, decremental responses in the right trapezius at 3, 5, and 10 Hz (top row) and the left trapezius (bottom row) at 3, 5, 10, and 20 Hz were detected in patient 2 **(B)**. In patient 3 **(C)**, RNS in the right trapezius (top row) at 3, 5, and 10 Hz and in the left trapezius (bottom row) at 3, 5, 10, and 20 Hz showed a decremental response. Repetitive nerve stimulation at low-frequency (3–5 Hz) and high-frequency (10–50 Hz), producing decremental responses >15 and 30%, respectively, were considered abnormal.

Among the three patients, motor and sensory nerve conduction velocities were normal. Needle EMG studies showed short-duration and low-amplitude motor unit potentials that are typical of myogenic changes. Additionally, dramatic decrements in RNS at low-frequency (3, 5 Hz) and high-frequency (10, 20, and 50 Hz) were observed in all three patients (Figure 1).

### Myopathological findings in muscle biopsy

**Figure 2:**
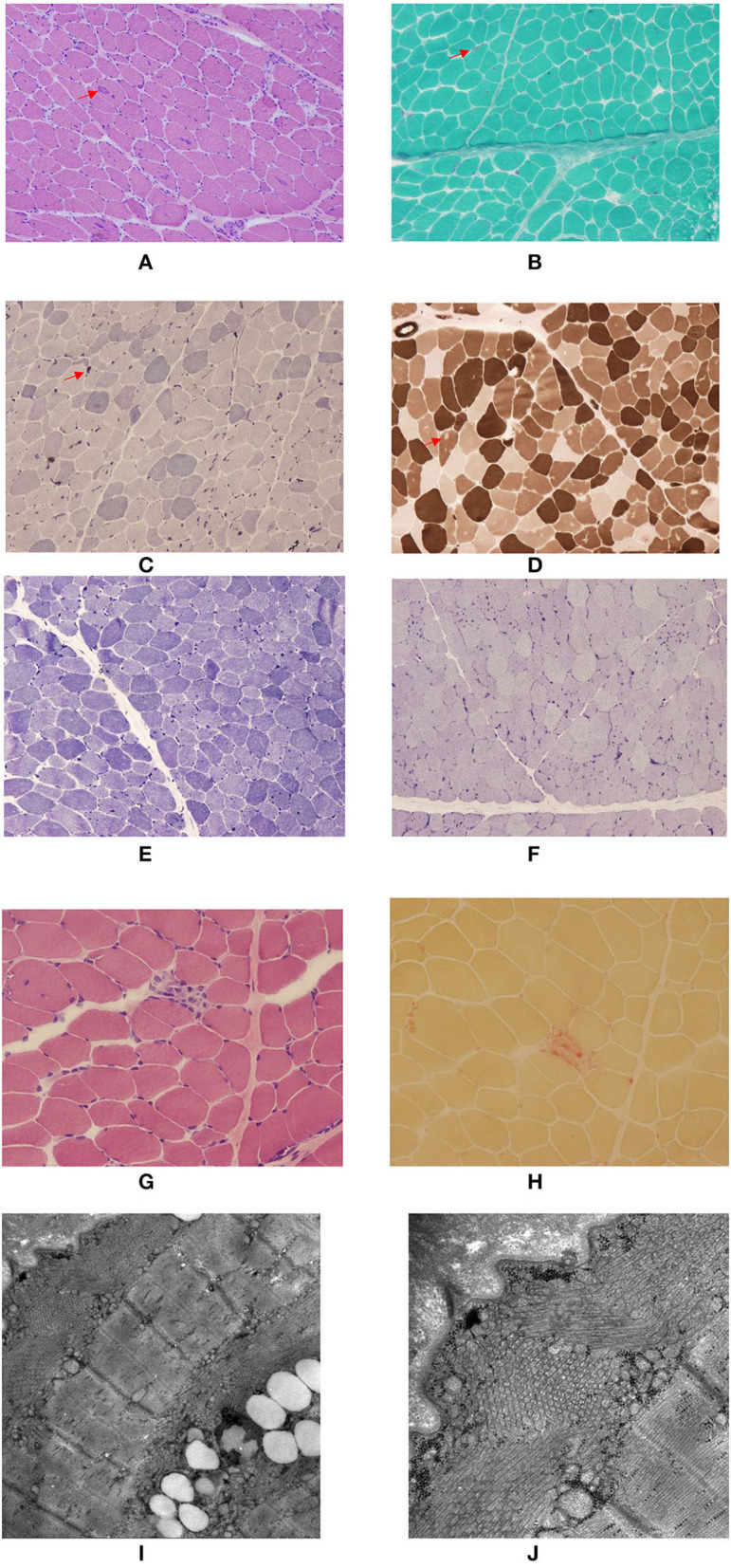
Myopathological findings of the patients with three GFPT1-related CMS from southwestern China. **(A**–**D)** Remarkable large and small tubular aggregates (TAs) (arrow head) located in both the subsarcolommal and inner cytoplasmic regions in patient 1. **(A)** H&E; **(B)**, Modified Gomori trichrome stain; **(C)** NADH-TR stain; **(D)** ATPase stain at pH 4.7 revealing the negatively stained TAs predominantly located in type 2 fibers. **(E,F)** Many small TAs located in both the subsarcolemmal and inner cytoplasmic regions in patient 2. **(E)** NADH-TR stain; **(F)** AMP stain. **(G,H)** A few TAs located in the subsarcolemmal region and focal fiber necrosis in patient 3. **(G)** H&E; **(H)** acid phosphatase stain. **(I,J)** Electron microscopy images of TAs in patient 1 and patient 2. **(I)** TAs located both beneath the sarcolemma and in the inner cytoplasmic region. **(J)** TAs in both transverse and longitudinal sections.

All three patients showed typical myogenic changes in muscle biopsy, such as fiber size variation of different degrees, occasional angular fibers, focal fiber necrosis, and occasional angular fibers. Additionally, TAs, predominantly in type 2 fibers, which were basophilic with H&E, red with the modified Gomori trichrome stain, and intensively blue with NADH-TR and AMP, reacted for acid phosphatase but were negative for SDH, COX, PAS, Sudan black, and myofibrillar ATPase reactions (Figures 2A–H). Electron microscopy images of TAs in patient 1 and patient 2 are shown in Figures 2I,J.

### Molecular genetic results

A homozygous missense mutation in exon 4 of GFPT1 (c.331C>T, p.R111C) (NM_002056.3) was identified in patients 1 and 2, which was confirmed by Sanger sequencing. Then, their asymptomatic parents were found to be heterozygous missense mutation carriers by Sanger sequencing (c.331C>T, p.R111C). Also, Sanger sequencing confirmed that one younger sister of patient 1, presenting with similar symptoms of proximal muscle weakness and cataract, was found to harbor the same homozygous GFPT1 mutation (c.331C>T, p.R111C) as patient 1.

A homozygous missense mutation in GFPT1 (c.44 C>T, p.T15M) was detected in patient 3 by targeted neuromuscular disorder gene panels and identified by Sanger sequencing from another famous tertiary diagnosis and treatment center, which is a university hospital in China. Due to adoption by her foster parents, the identification of mutations from her biological parents could not be conducted.

The detailed findings of clinical, laboratory, electrophysiological, myopathological, and molecular genetic characteristics of all three patients in our study are presented in Supplementary Table 1. In addition, globally, the clinical, laboratory, electrophysiological, and myopathological results of patients with LG-CMS with GFPT1 mutations are reviewed and summarized in Supplementary Table 2. Mutational variants in patients with GFPT1-related LG-CMS from different countries and ethnic populations are also summarized in Supplementary Table 3.

## Discussion

In our study, all three patients with GFPT1-related CMS presented with a typical limb-girdle pattern of muscle weakness, with no involvement of ocular, facial, bulbar, or respiratory muscles, a normal or mildly elevated serum creatine kinase, negative anti- AChRs, and anti-MuSK antibodies in two patients, a decremental response of compound muscle action potential (CMAP) amplitude on RNS at both low-frequency (3, 5 Hz) and high-frequency (10, 20, and 50 Hz), myogenic changes in electromyography, typical myopathological damages and TAs in muscle biopsy, homozygous missense mutations in GFPT1, and beneficial and sustained response to anticholinesterase inhibitor medication. All these findings were consistent with those of previously reported LG-CMS patients with GFPT1 mutations worldwide (6–27).

Similar to previous reports, patients 1 and 2 also displayed distal limb muscle weakness in addition to predominantly proximal limb involvement (6–8, 16, 18–20, 22, 23, 25). Most patients with GFPT1 mutations showed fluctuations in symptoms, including diurnal fluctuations, daytime-dependent fluctuations, day–day fluctuations, fluctuations over longer periods (8), or fluctuations from a few days to a few months (6). However, only diurnal fluctuations were observed in our three patients. In addition, it has been reported that exercise, fever, and infection could aggregate symptoms of muscle weakness (8), which was also found in our patients.

Retinal involvement as an additional feature has been reported in three patients with GFPT1 from two families: two having juvenile macular degeneration, and another having retinitis pigmentosa. Whether the retinal disease is associated with GFPT1 mutations, or a genetically distinct disorder is unclear (8). In our study, one of the younger sisters of patient 1, with the same homogenous GFPT1 missense mutations as patient 1, presented with congenital cataracts and prominent fluctuating and fatigable limb weakness. However, after a comprehensive examination by a specialized ophthalmologist, cataracts or other abnormalities of both eyes were not found in patient 1, indicating that cataracts did not co-segregate from the GFPT1 mutations. Therefore, we suggest that cataracts may be a genetically distinct disease entity that are independent of GFPT1-related CMS in this family.

A decremental response on RNS at 2–3 Hz has been found in GFPT1-related CMS from different countries (6, 8, 10–12, 14, 18, 20–22, 24–26). Although RNS at high frequency (10–50 Hz) was not performed routinely for patients with LG-CMS, only two previous studies on GFPT1-related CMS, performing repetitive nerve stimulation of the left ulnar nerve at 10 Hz and in the left trapezius, right trapezius, and right abductor digiti minimi muscle at 30 Hz, revealed no decrement (18, 25). However, in our three patients, repetitive nerve stimulations at both low and high frequencies showed a significant decrement in the amplitude of CMAP, especially in the trapezius, which is consistent with a previous report that indicated that to increase the accuracy, RNS should be carried out in proximal muscles if an LG-CMS is suspected (3). Our findings may suggest trapezius as a suitably targeted muscle for performing RNS in patients under the suspicion of GFPT1-related CMS.

In a previous study conducted by LoRusso and Iyadurai in 2017 (28), they reported a CMS patient with mutations in RAPSN, whose encoded protein acted to concentrate and anchor acetylcholine receptors in the postsynaptic membrane. The results of RNS showed significant decrements at higher frequencies (15 and 30 Hz), compared with the non-significant decrement at low frequencies, indicating the importance of a detailed electrodiagnostic testing in patients with suspected myasthenic syndrome (28). So, combining the results in our study, which found a significantly decremental response at both low- and high-frequency RNS in patients with GFPT1-related CMS, we further demonstrated the significance of detailed electrodiagnostic testing in patients with CMS, especially the high-frequency RNS. Seeking to characterize and understand these electrodiagnostic responses will help to further our knowledge of the neuromuscular junction (28). In addition, whether electrodiagnostic features of high-frequency RNS are specific features of some CMS subtypes (such as patients with GFPT1-related CMS in our study or patients with RAPSN-related CMS in the study by LoRusso and Iyadurai) and helpful for diagnosis deserved to be further studied, which were also greatly significant in our study. Unfortunately, like the study by LoRusso and Iyadurai (28), the exact pathophysiology underlying this finding remained unknown, which deserves further study in the future.

It is well-known that there can be an abnormal decrement in high-frequency RNS in severe myasthenia gravis, in contrast to no decrement of CMAP on high-frequency RNS in mild myasthenia gravis. Moreover, the response to high-frequency RNS can be normal in the mild postsynaptic block, while in the severe postsynaptic block, the high-frequency RNS can induce a decremental response and seemingly provide evidence for a severe postsynaptic block in our three patients. However, compared with previously reported patients, the severity of symptoms in our three patients was not very serious. Therefore, like myasthenia gravis, whether there was a correlation between high-frequency RNS and the severity of clinical symptoms in patients with GFPT1-related LG-CMS deserves a further study of high-frequency RNS in a larger sample of CMS patients with GFPT1 mutations from different countries.

In addition, as Figure 1 shows, as the frequency of stimulation increased, the amplitude or degree of decrement in CMAP seemed to be obvious among all three patients. A previous study found that a patient with SCN4A-related CMS, who had normal RNS at 2 Hz, showed a decremental response after a 5-min conditioning stimulation train at 10 Hz, suggesting that prolonged RNS at 10 Hz for 5 min should be performed to search for a defect of neuromuscular transmission in patients with a high index of suspicion for CMS who had normal RNS at 2 Hz (15). A similar phenomenon was also found in another two studies of patients with RAPSN-related CMS, in which abnormal decrement was only seen or more obviously at higher frequencies (28, 29). Therefore, our study further demonstrated that the frequency of stimulation should be increased gradually to help find evidence of neuromuscular transmission defects in patients with suspicion of CMS, especially when the low-frequency RNS was normal or showed an unapparent decrement, high-frequency RNS would be highly suggested.

TAs are characterized as more or less densely packed aggregates of vesicular or tubular membranes of variable forms and sizes thought to derive from the sarcoplasmic reticulum (8). They are believed to represent an adaptive mechanism to avoid rising intracellular levels of calcium in preventing muscle fibers from hypercontraction and necrosis (7, 22) TAs can be seen by light microscopy as dark inclusions in the NADH stain of muscle biopsies. A more specific way to identify TAs is by electron microscopy (EM) (8). In previously published reports, tubular aggregates were mainly detected in subsarcolemmal regions (NADH staining) (6, 8, 20), some TAs in EM (6, 8), and others both in the subsarcolemmal and inner cytoplasmic regions (22). In our study among our three patients, the clinical manifestations of patient 1 were the most serious, and the serum creatine kinase level was significantly elevated, patient 2 was moderate, and patient 3 was the mildest. Notably, the proportion of muscle fibers containing TAs, the regions (subsarcolemmal or inner cytoplasmic region, or both) where TAs were detected and the severity degree of non-specific myopathic findings on muscle biopsy seemed to have a positive relationship with the severity of clinical symptoms and the serum creatine kinase levels among three patients, which have never been reported previously. However, considering the relatively small sample in our study, this interesting phenomenon deserves close attention and identification in future studies.

Both GFPT1 missense mutations detected (c.331C>T, p.R111C; c.44C>T, p.T15M), located in the glutamine aminotransferase type-2 domain of the GFPT1 gene, have been reported in patients with GFPT1-related LG-CMS previously (6–8, 12, 16, 20). As shown in Supplementary Table 3, it was clear that two GFPT1 mutations, c.331C>T (p. Arg111Cys), and c.44C>T (p. Thr15Met), were detected in four and two out of six families in China, c.^*^22C>A in four out of twelve families in USA and two out of seven families in Spain, c.332G>A (p. Arg111His) in four out of nine families, and c.331C>T (p.Arg111Cys) in two out of nine families in France, suggesting that those mutations may be common mutations or hotspot mutations in those countries. Although different nucleic acids were affected by the c.331C>T and c.332G>A mutations, both mutations affected the same amino acid residue of Arg, confirming the hotspot status for mutation of this codon. Overall, c.331C>T (p. Arg111Cys), c.44C>T (p. Thr15Met), c.^*^22C>A, and c.332G>A (p. Arg111His) may be potential hotspot mutations in patients with GFPT1-related LG-CMS worldwide, deserving further study in larger samples of patients with GFPT1-related CMS.

## Conclusion

In conclusion, a dramatic decrement in high-frequency RNS, especially in the trapezius, was found in three patients with GFPT1-related CMS from southwestern China, which has never been reported, indicating that the trapezius may be a suitable targeted muscle for performing RNS in patients under the consideration of GFPT1-related CMS. Also, the location and degrees of TAs seemed to be related to the severity of clinical symptoms and the serum creatine kinase levels. Lastly, potential hotspot mutations in GFPT1 were found by reviewing all studies on GFPT1-related CMS. The findings of our study further expanded the phenotypic spectrum of GFPT1-related LG-CMS and improved our understanding of this rare muscle disorder in clinical practice.

## Data availability statement

The original and analyzed data in this study are available from the corresponding author on reasonable request.

## Ethics statement

The present study was approved by the Institutional Ethics Committee of West China Hospital of Sichuan University (approval 2020-842). Written informed consent was obtained from the patients or patients' legal guardian for participating in the study and publication of this paper.

## Author contributions

RA, HC, SL, and YL performed the material preparation, data collection, and analysis. RA wrote the first draft of the manuscript. YX and CH designed the research project and revised the manuscript. All authors contributed to the study conception, design, commented on previous versions of the manuscript, and approved the final manuscript.

## Funding

This study was supported by the Platform of Resource Collection and Standardized Diagnosis and Treatment for Neurogenetic Degeneration Diseases (2019JDPT0015 to YX), the Post-Doctor Research Project, West China Hospital, Sichuan University (2020HXBH145 to RA), the Key Research and Development Projects, Department of Science and Technology of Sichuan Province (2021YFS0223 to RA), the National Natural Science Foundation (81902287 to YL), and Cooperative Development Project Fund of West China Hospital, Sichuan University (hx-h2107188 to CH).

## Conflict of interest

The authors declare that the research was conducted in the absence of any commercial or financial relationships that could be construed as a potential conflict of interest.

## Publisher's note

All claims expressed in this article are solely those of the authors and do not necessarily represent those of their affiliated organizations, or those of the publisher, the editors and the reviewers. Any product that may be evaluated in this article, or claim that may be made by its manufacturer, is not guaranteed or endorsed by the publisher.
