# Supplementary material for: Abnormal decrement on high-frequency repetitive nerve stimulation in congenital myasthenic syndrome with GFPT1 mutations and review of literature
Source: Front Neurol. 2022 Sep 15;13:926786. doi: 10.3389/fneur.2022.926786 (PMC9520358; doi:10.3389/fneur.2022.926786)
Supplement: Supplementary file 1 [file Data_Sheet_1.PDF]

Supplement-table 1 Clinical, laboratory, electrophysiological, myopathological and molecular genetic characteristics of patients with congenital myasthenic syndrome with tubular aggregates with GFPT1 mutations from southwestern China

Patient	patient 1	patient 2	patient 3
**Consanguineous family(n)**	Yes	No	NA
**Family history (n)**	Yes, a younger sister, aged 29, suffered with similar symptoms for more than ten years.	No	NA
**Gender (M, F)**	M	M	F
**Age(y)**	32	14	15
**Onset age (y)**	13	9	6
**Symptom at onset**	Difficulty of climbing mountain，alleviate after rest	Waddling gait	Falling easily, abnormal posture
**Motor and mental milestones**	Normal	Normal	Normal
**Weakness distribution**			
Ptosis/ophthalmoplegia	No/No	No/No	No/No
Facial/bulbar/respiratory muscle	No/No/No	No/No/No	No/No/No
Proximal limbs muscle	Yes	Yes	Yes

Distal limbs muscle	Yes	Yes	No
**Predominant LG weakness**	Yes	Yes	Yes
**Muscle atrophy**	No	No	No
**MRC**	44	47	49
**Fluctuations**	Yes	Yes	Yes
**Fatigability**	Yes	Yes	Yes
**Aggravating factors**	fever, infection, exercise	exercise	fever, infection, exercise
**Neostigmine test**	ND	Positive	ND
**Serum CK level**	Mildly elevated (335, normal range:19-226 IU/L)	Normal (203, normal range: 19-226 IU/L)	Normal (87, normal range: 20-140 IU/L)
**Anti- AChRs、anti-Musk antibodies**	Negative	Negative	ND
**Nerve conduction study**	Normal	Normal	Normal
**Decrement on RNS at 3-5 Hz and 10-50 HZ)**	A dramatic decrement in the right trapezius on RNS at 3, 5, 10, 20HZ and in the right abductor hallucis at 5, 20, 50HZ.	A decremental response in the right abductor pollicis brevis at 3, 5HZ, in the right trapezius at 3, 5, 10HZ and in the left trapezius at 3, 5, 10,20HZ.	A decrement on RNS in the right tibialis anterior at 3, 5HZ, in the right trapezius at 3, 5, 10HZ and in the left trapezius at 3, 5, 10, 20HZ.

**EMG (Myopathic changes)**	Yes	Yes	Yes
**TAs in muscle biopsy**	In 35% fibers, both large and small, both in the subsarcolommal and inner cytoplasmic regions in LM, also in EM	In 20% fibers, small, both in the subsarcolommal and inner cytoplasmic regions in LM， also in EM.	In 10% fibers, mostly small, occasionally large, only in the subsarcolommal region in LM, not in EM
**Other myopathological findings**	Moderate fiber size variation, focal fiber necrosis, occasional nuclear clumps and angulated fibers, mild endomysial fibrosis.	Mild to moderate fiber size variation, occasional angular fibers	Mild fiber size variation, focal fiber necrosis, occasional angular fibers
**Nucleotidic mutations (GFPT1)**	c.331C>T, homozygous	c.331C>T, homozygous	c.44 C>T, homozygous
**Protein variations**	p.R111C	p.R111C	p.T15M
**Identified in biological parents**	Yes, both in a heterozygous state	Yes, both in a heterozygous state	NA
**Clinical course (Two-year follow-up)**	Stable	Stable	Stable
**Therapy response to AChE inhibitors**	pyridostigmine, 60 mg, three times a day, a favorable and sustained improvement	pyridostigmine, 60 mg, three times a day, a favorable and sustained improvement	Pyridostigmine,60 mg, once a day, a favorable and sustained improvement

Abbreviations: n, number; M, male; F, female; y, years; NA, not available; LG, Limb-girdle; MRC, Medical Research Council; ND, not done; CK, creatine kinase; AChRs: acetylcholine receptors; MUSK: musclespecific tyrosine kinase; RNS, repetitive nerve stimulation; EMG, electromyography; TA, tubular aggregates; LM: light microscopy; EM: electron microscopy; GFPT1: glutamine-fructose-6-phosphate transaminase 1; AChE, acetylcholinesterase.

The amplitude of compound muscle action potential (CMAP) on RNS at both low-frequency (3, 5 HZ) and high-frequency (10, 20, 50 HZ), which decreased by more than 15% and 30%, respectively, are considered abnormal.

The numbering of nucleotides and amino acids follows NM_002056.3 and NP_002047.2, which represent the short, ubiquitous isoform of GFPT1.

Therapeutic response to AChE inhibitors: the clinical improvement is assessed by patient or their legal guardian reported outcomes or documented by clinical physical examinations.

Supplement-table 2 Clinical, laboratory, electrophysiological and myopathological characteristics of patients with congenital myasthenic syndrome with tubular aggregates due to mutations in GFPT1 worldwide

Country [Ref]	USA[10,17,19]	Iran[4,7]	Turkey[4,7,18]	Libya[4,7]	Sweden[4,7]	Spain[4,7,8,9,22,26]	Germany[4,7]	UK[4,6,7]	Senegal[4,7]	Italy[4,7]	Malta[4,7]
**Family (n)**	12	1	2	1	1	7	2	2	1	1	1
**Consanguineous family(n)**	2(11)	1(1)	2(2)	1(1)	0(1)	2(4)	0(2)	0(2)	1(1)	1(1)	0(1)
**Family history (n)**	5(12)	1(1)	0(1)	1(1)	0(1)	2(4)	0(2)	0(2)	0(1)	1(1)	1(1)
**Patients (n)**	12	2	2	5	1	10	2	2	1	2	2
**Gender (M, F)**	8/12(M), 4/12(F)	2(M)	1(M),1(F)	2(M),3(F)	1(M)	4(M),6(F)	1(M),1(F)	2(M)	1(F)	2(M)	1(M),1(F)
**Onset age (y)**	in utero-19	6	6, NM	6	First decade	birth-40s	5,13	8,6	1	7,10	8,7
**Symptom at onset**											
Ptosis		0(2)	0(1)	0(5)	0(1)	0(6)	0(2)	0(2)	0(1)	0(2)	0(2)
Limb muscle weakness	1(1)	2(2)	1(1)	5(5)	1(1)	7(7)	1(2)		1(1)	2(2)	
Fatigability	1(1)	2(2)	1(1)	5(5)			1(2)	1(2)			2(2)
Fall						2(6)		1(2)	1(1)		1(2)
Waddling gait											
Difficulties in running	1(1)										1(2)
Hypotonia, poor sucking						1(1)					
Motor milestones delay											
**Delayed Motor milestones**	1(12)	0(2)	0(2)	0(5)	0(1)	4(9)	0(2)	1(2)	0(1)	0(2)	1(2)

**Weakness distribution**											
Ptosis/OPH	-/-	-/-	-/-	-/-	-/-	0(8)/0(8)	-/-	1(2)/-	-/-	-/-	-/-
F/B/C/R	2(2)/1(1)/1(1)/2(12)	2(2)/-/-/-	-/-/1(1)/0(1)	-/-/-/-	-/-/-/-	2(8)/1(8)/5(8)/1(8)	-/-/-/-	2(2)/-/1(2)/1(2)	-/-/-/-	-/-/-/-	-/-/-/-
Proximal limbs	12(12)	2(2)	2(2)	5(5)	1(1)	8(8)	2(2)	2(2)	1(1)	2(2)	2(2)
Distal limbs	11(11)	2(2)	1(2)	0(5)	0(1)	5(8)	0(2)	2(2)	0(1)	0(2)	0(2)
**Predominant LG weakness**	8(12)	2(2)	2(2)	5(5)	1(1)	6(6)	2(2)	2(2)	1(1)	2(2)	2(2)
**Muscle atrophy**		2(2)	1(2)	5(5)	0(1)	4(7)	0(2)	0(2)	0(1)	0(2)	0(2)
**Additional features**		juvenile macular degeneration2(2)	Mild scoliosis, pes cavus deformity1(1)	-	-	Miopathic facies,prognathism,1(9)/ Scoliosis,3(9)	-	1(2) retinitis pigmentosa	-	-	2 (2) learning difficulties
**Fluctuations**	1(11)	2(2)	1(1)	ND	1(1)	3(6)	2(2)	1(2)	1(1)	0(2)	2(2)
**Fatigability**	1(1)					1(1)		2(2)			
**Aggravating factors**											
Exercise				5(5)							
Menstruation											
Heat, infection		2(2)	1(1)	5(5)				1(2)			
**Clinical course**											
Stable						1(6)	1(2)				
Improving		2(2)						1(2)			
Worsening			1(1)		1(1)	6(7)	1(2)	1(2)	1(1)	1(1)	2(2)
Progressive	10(11)					1(1)					
**Serum CK level**											

normal	6(7)		1(2)	5(5)		4(6)	1(2)		1(1)		
elevated	1(7)	2(2)	1(2)		1(1)	2(6)	1(2)	2(2)		2(2)	2(2)
**Negative Anti-AChR antibody**	11(11)	2(2)	1(1)	5(5)	1(1)	6(6)	2(2)	2(2)	1(1)	2(2)	2(2)
**Decrement on RNS at 2-3 Hz**	12(12)	2(2)	1(1)	5(5)	1(1)	6(7)	2(2)	2(2)	1(1)	2(2)	0(1)
**Decrement on RNS at 10-50 Hz**											
Normal decrement	10HZ, 1(1)										
**Normal nerve conduction**	11(11)		1(1)			Low compound muscle action potential, 1(1)					
**EMG**											
normal											
myogenic changes	1(11)	1(1)	1(1)	5(5)	1(1)	6(6)	1(1)		1(1)	2(2)	
**SFEMG (increased jitter)**			1(1)		1(1)	5(5)		2(2)			
**TA in muscle biopsy**	7(9)	1(1)	1(1)	1(1)	1(1)	3(5)	2(2)	2(2)	0(1)	2(2)	0(2)
**Other myopathological findings**	Type 1 fiber preponderance, 3(9)/ Small vacuoles, 3 (9)/ regenerating and necrotic	Fiber size variation, type 1 fibre predominance, round or	Chronic myopathic changes,1(2), dystrophic pattern,1(2)	-	Increased fiber size variability Frequent fibres with internalized nuclei and	Unspecific or mild myopathic changes, 4(5)/ type 1 fibres predominan	Unspecific myopathic changes,1(2)	Muscle atrophy, multiple internal nuclei, Vacuoles, denervat	Uneven oxidative staining, accumulation of Mitochondria,1(1)	-	Fibre size variability,2(2)/type 2 predominance with occasional core-like areas and

	fibers1(9)/fiber-type grouping and small fibers, 3(9).	angular fibres,1(2)			autophagic vacuoles,1(1)	ce and ragged red-like fibres,2(5)		ion features, 1(2)			subtle uneven staining,1(2)
**Positive Therapy Response**											
A	11(12)	2(2)	1(1)	5(5)	1(1)	6(6)	2(2)	2(2)	1(1)	2(2)	2(2)
3,4 DAP	5(6)				1(1)	3(4)		2(2)	1(1)		1(2)
S	1(1)										
E	1(1)										

**Country** Ref	**Australia**[16]	**New Zealand**[14]	**Israel**[12]	**France**[5]	**Korea**[21]	**Netherlands**[20]	**Japan**[25]	**India**[13]	**Mexico**[9]	**China**[11,23,24] **and our study**
**Family (n)**	1	1	1	9	1	2	1	3	1	13
**Consanguineous family(n)**			0(1)	1(9)		2(2)	0(1)	1(3)		1(11)
**Family history (n)**				2(9)	0(1)	2(2)	0(1)	1(3)	1(1)	2(13)
**Patients (n)**	1	1	1	11	1	4	1	3	2	14
**Gender (M: F)**	F				M	4(M)	F	M (2), F (1)	M (2)	9(M),5(F)
**Onset age (y)**			5	1-24	13	Neonatal-5y	1.5	1.5-11	Birth,2(2)	Birth-17
**Symptom at onset**								NM		
Ptosis										0(14)
Limb muscle weakness				8(11)	1(1)	2(4)				10(14)

Fatigability			7(11)	1(1)					2(14)
Fall									1(14)
Waddling gait									2(14)
Difficulties in running						1(1)			0(14)
Hypotonia, poor sucking or dysphagia								1(1)	
Motor milestones delay					2(4)				
**Delayed Motor milestones**	1(1)		1(11)	0(1)	4(4)	0(1)	2(3)		3(11)
**Weakness distribution**									
Ptosis/OPH		-/-	4(11)/-	-/-		-/-	-/-	2(2)/NM	1(14)/-
F/B/C/R	1/NM/NM/NM		1(11)/1(11)/NM/NM	-/-/NM/NM	NM/NM/1(4)/2(4)	1(1)/-/1(1)/-	-/-/-/-	2(2)/2(2)/2(2)/2(2)	-/1(12)/1(12)/1(12)
Proximal limbs			11(11)	1(1)	4(4)	1(1)	3(3)	2(2)	14(14)
Distal limbs			11(11)	1(1)	1(4)	1(1)	0(3)	2(2)	3(12)
**Predominant LG weakness**			11(11)	1(1)	4(4)	1(1)	3(3)		13(14)
**Muscle atrophy**				0(1)	1(4)	1(1)	0(3)	1(1)	1(12)
**Additional features**	Congenital hypotonia, contracture, scoliosis,1(1)		flat feet 2(11)/ Achilles retractions 2(11)	mild contractures in left hip and ankle joints	Myopia, bilateral retinoschisis 2(2), hypotonic and tachypnea at birth,1(4), neonatal respiratory distress; intraventricular hemorrhage,1(4)			bilateral cryptorchidism, micrognathia, a high arched palate without evidence of cleft, head lag1(2)/ broad nasal bridge, micrognathia, a	

								tented upper<br>lip, high and<br>narrow palate,<br>slightly wide<br>set<br>nipples, and<br>descended<br>testes,1(2)	
**Fluctuations**			11(11)			1(1)			12(13)
**Fatigability**				1(1)		1(1)	3(3)		6(6)
**Aggravating factors**									
Exercise									5(5)
Menstruation						1(1)			1(1)
Heat, infection					2(4)				3(3)
**Clinical course**									
Stable			1(11)			1(1)			5(5)
Improving			2(11)	1(1)	2(2)				
Worsening			7(11)						
Progressive									
**Serum CK level**									
normal	1(1)		4(11)		2(2)	1(1)		1(1)	5(13)
elevated		1(1)	7(11)	1(1)					8(13)
**Negative Anti-AChR Ab**				1(1)		1(1)	3(3)		5(5)

**Brain MRI**				Diffuse cerebral white matter abnormalities, selective involvement of the middle blade of the corpus callosum, 4(4), cerebellar white matter hyperintensity,2(2)				Normal,3(3)
**Decrement on RNS at 2-3 Hz**	only neuromuscular junction dysfunction mentioned	11(11)	1(1)	2(2)	1(1)	decremental response, stimulation frequency not mentioned, 3(3).	1(1)	13(13)
**Decrement on RNS at 10-50Hz**								
Normal								1(1)(30HZ)
decrement								3/3(10,20,50 HZ)
**Normal nerve conduction**		11(11)	1(1)	1(1)				4(4)

**EMG**									
normal									1(12)
Myogenic changes		Fibrillation potentials	8(11)	1(1)	1(1)	1(1)			11(12)
**SFEMG (increased jitter)**				1(1)				1(1)	1(1)
**TA in muscle biopsy**		1(1)	6(6)	1(1)		1(1)		0(2)	10(12)
**Other myopathological findings**	dystrophic with degenerating and regenerating fibres,1(1)				focal areas of degeneration, regeneration, or necrosis, ragged red fibers.2(2)	mild myopath ic changes		abnormally small muscle fibers, 1(2), mild necrotizing myopathy with extensive auto-phagic vacuolar pathology,1(2)	Rimmed vacuoles (1/11), Mild changes or non-specific myopathies (6/11), angulated fibers(4/11)
**Positive Therapy Response**									
A	1(1)	1(1)	9(9)	1(1)	2(2)	1(1)	1(1)	1(1)	12(12)
3,4 DAP			9(9)					1(2)	
S		1(1)					0(1)		1(1)
E						1(1)			

Abbreviations: Abbreviations: Ref, reference; n, number; the number in the round blanket was the number of patients examined, the number outside the round brackets was the number of patients with this symptoms, signs or findings; M, male; F, female; y, years; OPH, Ophthalmoplegia; F/B/C/R, Facial/Bulbar/Cervical/Respiratory; NM, not mentioned; LG, Limb-girdle; CK, creatine kinase; AchR, acetylcholine

receptors; Ab, antibody; MRI, magnetic resonance imaging; RNS, repetitive nerve stimulation; EMG, electromyography; SFEMG, single-fiber electromyography; TA, Tubular aggregates; A, acetylcholine esterase inhibitor; S, Salbutol; E, Ephedrine.

-, absent of symptom or sign; all the blank spaces in the table means not mentioned or not done in the literature.

Supplement-table 3 Mutational variants in patients with congenital myasthenic syndrome with tubular aggregates due to mutations in GFPT1 worldwide

**Country** [Ref]	Number	Mutations in GFPT1	Common mutations or hotspot mutations
China[11,23,24] and our study	14(13)	c.331C>T, p.Arg111Cys(4)/c.1648G>A, p.Ala550Thr/c.1949T>C, p.Val650Ala/c.44C>T, p.Thr15Met(2)/c.1100A＞G, p.Tyr367Cys/c.1690G>T, p.Gly564Cys/c.736A>T, p.Arg246*/c.1927A>C, p.Thr643Pro/c.76G＞A, p.Gly26Ser/c.871G＞A, p.Val291Ile/c.2029C>T, p.His677Thr/ c.462G > C, p.Lys154Asn/c.1088A > G, p.Asn363Ser/c.14 T>A, p.F5Y/c.581 T>C, p.F194S.	c.331C>T, p.Arg111Cys(4)/ c.44C>T, p.Thr15Met(2)
USA[10,17,19]	12(12)	c.1700-1716dup17, p.Gly573LeufsX9/c.*22C>A(4)/c.1634G>C, p.Arg545Pro/c.606-8A>G/c.338A>G, p.Asp113Gly/c.1475T>C,p.Met492Thr/c.49C>T,p.Arg17X/c.686-2A>G/c.910C>T, p.Arg304X/c.331C>T,p.Arg111Cys/c.1049C>T,	c.*22C>A(4)

		p.Thr350Ile/c.1337delA,p.Ile446SerfsX41/c.1_2del2,p.Met1fsX2/c.44C>T,p.Thr15Met / c.2002G>A,p.Val668Ile/c.821T>C; p.Ile274Thr/c.41G>A,p.ARg14Gln.	
UK[4,6,7]	2(2)	c.1154G>A,p.R385H/c.1301, p.R434H/ c.44C>T,p.T15M/c.1486C>T, p.R496W	
Turkey[4,7,18]	2(2)	c.719G>A, p.Trp240X/c.686-2A>G.	
Spain[4,7,8,9,22,26]	10(7)	c.221A>C, p.His74Pro/c.686-2A>G (IVS8-2A>G)/c.686dupC, p.Arg230Ter/ c.1475T>C, p.M492T/ c.*22C>A(2)/c.1472T>C,p.Met491Thr/ c.1278_1281dup, p.Asp428fs.	c.*22C>A (2)
Australia[16]	1(1)	g.69581446T>C, c.686-2A>G/c.1072A>G, p.(Met358Val).	
New Zealand[14]		NM	
Israel[12]	1(1)	c.35T>C, p.Val12Ala/ c.1103G>A, p.Arg368His.	
France[5]	11(9)	c.207G>A,p.Gly39_Lys75delinsGlu/c.332G>A,p.Arg111His(4)/c.331C>T, p.Arg111Cys(2)/c.1174A>C, p.Thr392Pro/ c.1496T>G,p.Met499Arg/c.2002-1G>C/c.*22C>A/ c.949A>T, p.Met317Leu/c.44C>T,p.Thr15Met/c.739C>G,	c.332G>A,p.Arg111His(4)/c.331C>T, p.Arg111Cys(2)

		p.Leu247Val/c.1882A>G, p.Ile628Val/c.(955 + 1956-1)<br>(1051 + 11052-1)del,p.Gly319Thr3<br>50del**
Korea[21]	1(1)	c.766C>G, p.E256Q/ c.1496C>T,p.M499T
Netherlands[20]	4(2)	c.41G>T,p.Arg14Leu(1)/c.452C>A, p.Thr151Lys(1)
Japan[25]	1(1)	c.722_723insG,p.G241fs
India[13]	3(3)	c.156A>G, p.N53S/c.266T>C, p.L89P/c.540A>G, p.Q180Q/c.1421G>A, p.R474Q.
Mexico[9]	2(1)	c.686dupC,p.Arg230Ter(1)
Iran[4,7]	2(1)	c.1042G>T, p.D348Y(1)
Libya[4,7]	5(1)	c.331C>T, p.Arg111Cys(1)
Sweden[4,7]	1(1)	c.222_223insA,p.Gln76fs/ c.331C>T, p.Arg111Cys
Germany[4,7]	2(2)	c.128A>T,p.Asp43Val/c.362T>C, p.Ile121Thr/ c.595G>T, p.Val199Phe/c.*22C>A
Senegal[4,7]	1(1)	c.1534C>T, p.Arg512Trp

Italy[4,7]	1(1)	c.43A>G,p.Thr15Ala/c.621_622 del, p.Leu208fs
Malta[4,7]	2(1)	c.1472T>C,p.M491T/c.714_715insA

Ref: reference; NM: not mentioned. The number in the round brackets means the total number of family in the country, the number outside the round brackets means the total number of patients involved.

Ref 5, 15: The Nucleotide numbering of cDNA was based on GeneBank accession number NM_002056.3 for the short isoform and NM_00124710.1 for the muscle-specific long isoform,

Ref 26: The Nucleotide numbering of cDNA was based on GeneBank accession number NM_002056 for the short isoform.

Ref 14,18, 25: The Nucleotide numbering of cDNA was based on GeneBank accession number NM_001244710.1.

Ref 12: The transcripts of GFPT1 ENST00000357308 was used for the annotation of variants.
